# Patchy Bubble-Propelled Colloids at Interfaces

**DOI:** 10.1002/admi.202300226

**Published:** 2023-08-04

**Authors:** David P. Rivas, Max Sokolich, Harrison Muller, Sambeeta Das

**Affiliations:** Department of Mechanical Engineering, University of Delaware, 130 Academy Street, Newark, DE 19716, USA

**Keywords:** active colloids, bubble-propelled, interface, micro-assembly, micromanipulation, microrobots

## Abstract

Liquid–liquid or liquid–air interfaces provide interesting environments to study colloids and are ubiquitous in nature and industry, as well as relevant in applications involving emulsions and foams. They present a particularly intriguing environment for studying active particles which exhibit a host of phenomena not seen in passive systems. Active particles can also provide on-demand controllability that greatly expands their use in future applications. However, research on active particles at interfaces is relatively rare compared to those at solid surfaces or in the bulk. Here, magnetically steerable active colloids at liquid–air interfaces that self-propel by bubble production via the catalytic decomposition of chemical fuel in the liquid medium is presented. The bubble formation and dynamics of “patchy” colloids with a patch of catalytic coating on their surface is investigated and compared to more traditional Janus colloids with a hemispherical coating. The patchy colloids tend to produce smaller bubbles and undergo smoother motion which makes them beneficial for applications such as precise micro-manipulation. This is demonstrated by manipulating and assembling patterns of passive spheres on a substrate as well as at an air–liquid interface. The propulsion and bubble formation of both the Janus and patchy colloids is characterized and it is found that previously proposed theories are insufficient to fully describe their motion and bubble bursting mechanism. Additionally, the colloids, which reside at the air–liquid interface, demonstrate novel interfacial positive gravitaxis towards the droplet edges which is attributed to a torque resulting from opposing downward and buoyant forces on the colloids.

## 1. Introduction

The study of active particles at liquid–liquid and air–liquid interfaces involves many unique considerations compared to motion near a substrate or in the bulk. Particles at interfaces experience different viscous drag and/or chemical distributions while also introducing additional relevant forces such as capillary or surface-tension based forces.^[1]^ Interfaces also trap active particles in a quasi-2D space, which can result in considerably different behavior than that in the bulk. Interfaces are ubiquitous in nature and industry and therefore are important environments for studying the behavior of active colloids due to their potential relevance in future applications. Some examples of studies of active particles at interfaces include collections of bacteria^[2]^ or chemically fueled self-phoretically propelled Janus particles.^[3]^ Aside from few initial studies, there has been little research on active colloids at air–liquid and liquid–liquid interfaces compared to the bulk or at solid surfaces. Therefore, it is of importance to study the behavior of active colloids in these unique environments. Particles at interfaces of bubbles are also relevant in applications such as emulsions and foams as well as in the development of new materials.^[1]^

Bubble-producing colloids that catalyze fuel in their medium have been shown to self-propel and be used for breaking up of biofilms^[4]^ or pushing objects.^[5]^ Microscale colloids that can carry out tasks such as these are known as microrobots.^[6]^ Microrobots show promise in biomedical and engineering applications such as drug delivery, environmental remediation, enhanced biofilm removal, sensing, and micro-manipulation.^[7–16]^ A catalytic microrobot, a type of chemically driven microrobot, is powered by the chemical decomposition of a fuel source present in the medium.^[17]^ Two common modes of propulsion for such catalytic microrobots rely on self-phoresis or bubble generation.^[18–22]^ Bubble-propelled microrobots have the advantage of being relatively fast and powerful compared to their self-phoretic counterparts,^[ 19,23]^ features that are advantageous for many of the aforementioned applications. Specifically, these properties are particularly useful in tasks such as transport of cargo, rapid remediation, or microassembly. However, there have been limited examples of precise control and manipulation using bubble-propelled microrobots,^[5,24]^ as well as relatively few studies on their dynamics and bubble formation/dissolution process.^[21,22,25,26]^ There have also been limited studies on the temperature dependence of microrobot dynamics,^[27]^ particularly at values near room temperature. Exploring the temperature dependence of their dynamics provides insight into how these changes effect bubble generation and motion in realistic environmental conditions. It also is relevant to the use of microrobots as sensors to reveal properties of their environment.

Motion driven by self-phoretic effects, such as selfelectrophoresis or self-diffusiophoresis, derive from an induced concentration gradient of either ionic or neutral molecules, respectively. This gradient can form around particles with an anisotropic distribution of catalyst material on the particle’s surface. A typical example of this is a Janus spherical colloid in which one hemisphere of the sphere is coated in platinum. The platinum catalyzes hydrogen peroxide to form water and oxygen, which results in a concentration buildup of molecules around the colloid. In bubble-driven motion, the concentration of oxygen reaches saturation in the medium, leading to a bubble nucleating and growing.^[19]^ The motion of the colloid in this case is due party to the growth of the bubble, the hydrodynamic flows created upon its dissolution, or pressure differentials.^[21,22,26]^

A type of bubble-propelled machine that has been extensively studied is a microjet, a hollow tubular microrobot that produces bubbles inside the tube and ejects them out one end, propelling it toward the other.^[25,26]^ Microjets typically have lengths of 50–100 μm, diameters of 5–10 μm, and speeds of 300–3000 μms^−1^.^[19]^ This type of microrobot was previously used to transport spheres and manipulate micro-sheets at the liquid–air interface, but lacked precise magnetic control.^[5]^ One disadvantage of the microjet is its more complex fabrication process compared to spherically shaped Janus particles, which can be made using simple physical vapor deposition procedures.

For a spherical Janus catalytic colloid, self-phoretic motion generally occurs when the diameter of the colloid is less than about 10 μm.^[22]^ For larger diameters, bubbles begin to form at their surface due to the greater concentration of reactant products. As previously mentioned, self-phoretic microrobots move at much slower speeds and are significantly less powerful than bubble-propelled microrobots, making them less ideal for applications requiring manipulation of objects at the microscale, such as microassembly. For example, bubble-propelled microrobots are capable of an order of magnitude larger velocity, orders of magnitude increase in generated propulsion force, and much higher efficiency.^[23]^

Here, we study the motion and bubble generation of Janus and “patchy” colloids. The patchy colloids have a smaller area of surface coating of catalytic material, which we find results in generally smaller bubble size production. The patchy microspheres that produce bubble sizes of ≲25 μm have a smoother motion that lacks the forward/backward cyclical motion present in the case of larger bubble sizes that have been studied previously.^[22]^ Aside from the interesting observations of their difference in bubble generation, the smaller bubble sizes and smoother motion of these colloids makes them well suited for precise manipulation. Therefore, we use the patchy colloids to selectively manipulate and assemble individual particles into patterns on the solid substrate as well as at the air–liquid interface. We also compare the dynamics and bubble growth process of both the hemispherically coated Janus as well as the “patchy” bubble propelled colloids as a function of hydrogen peroxide concentration and temperature. Aside from micromanipulation, moving at the liquid–air interface makes the bubble-propelled colloids potentially useful tools in applications at interfaces such as in environmental remediation or localized interfacial biofilm removal.

## 2. Results and Discussion

### 2.1. Patchy Colloids

**Figure 1.**
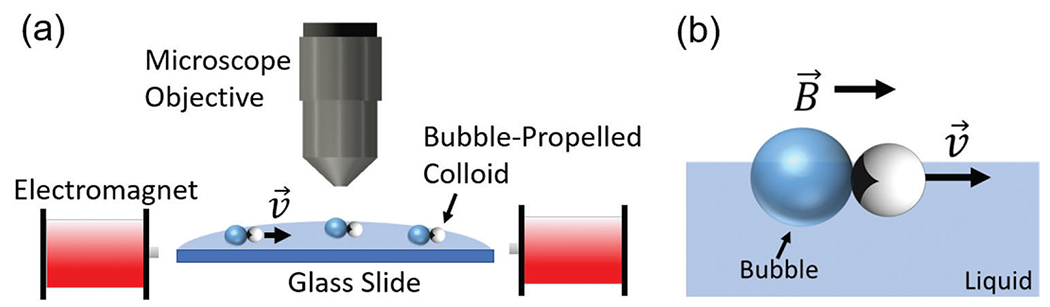
a) Experimental schematic showing the buoyant bubble-propelled colloids in hydrogen peroxide solution and two of the four electromagnets used to steer them. b) A schematic of a patchy bubble-propelled colloid steered by a magnetic field.

**Figure 2.**
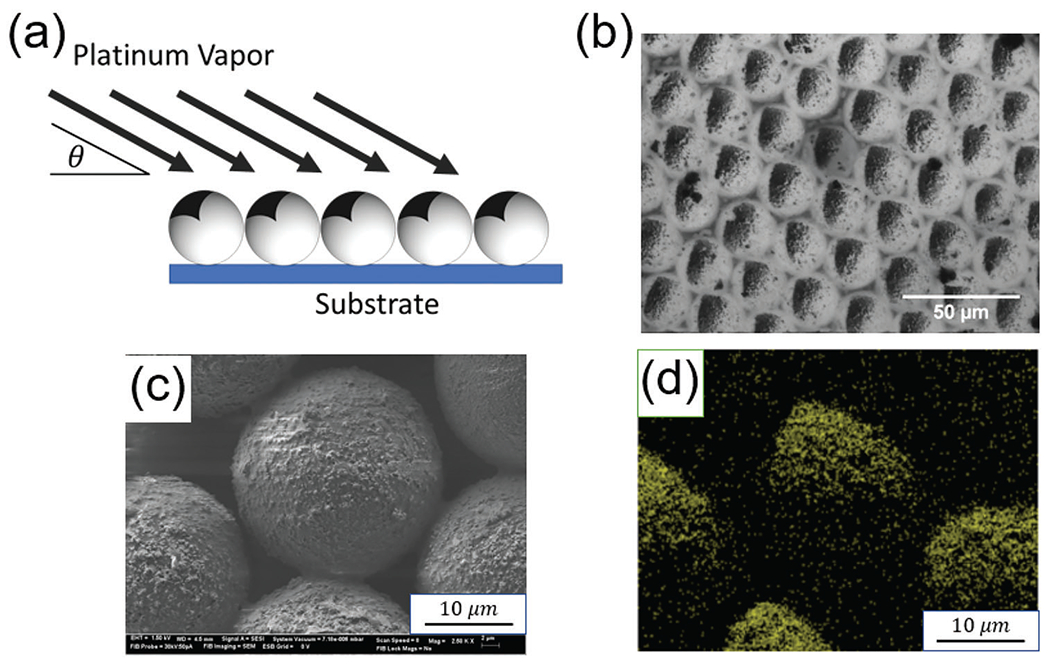
a) A sketch of the glancing-angle deposition onto the densely packed spherical colloids. The angle of deposition is denoted by *θ*. b) A fluorescent image of the spheres after the glancing-angle deposition of platinum on their surface. c) SEM image of a patchy colloid. d) EDX image of patchy colloid showing the distribution of Pt on the surface.

Figure 1a shows a schematic of the experimental setup. The colloids were placed in a solution of hydrogen peroxide, which was spread onto a hydrophilic glass slide. Electromagnets were used to steer the magnetic colloids, as depicted in Figure 1b. The active colloids were fabricated using e-beam deposition, as depicted in Figure 2a. Paramagnetic spheres were coated with platinum at an angle of *θ* = 10° to create the “patchy” colloids. The Janus active colloids that were used as a comparison were first coated with iron followed by platinum at a deposition angle of *θ* = 90°. Iron was deposited to make the microrobots more magnetic and therefore more easily steered, which was useful for maintaining linear motion of the most active colloids (see Figure 2). Figure 2b shows a fluorescence image displaying the platinum coating on the surface of the patchy colloids and Figure 2c displays an SEM image of the colloids. The surface coverage of the patchy colloids is approximately 1/4 of that of the hemispherically coated colloids.^[28]^ More details on the fabrication and experimental procedures can be found in the Experimental section.

We study both the patchy and Janus active colloids at hydrogen peroxide concentrations of 10, 15, and 30% (w/v), as well as at temperatures of 19.4, 21.7, 24.4, and 26.7 degrees Celsius. Experiments done at the various hydrogen peroxide concentrations were conducted at a temperature of approximately 24 °C and the temperature controlled experiments were all conducted at a hydrogen peroxide concentration of 30%.

The colloids were suspended in a solution of hydrogen peroxide and then viewed through a microscope on a glass slide. The platinum on the colloid surface acts as a catalyst of the hydrogen peroxide solution, resulting in the formation of oxygen bubbles. The bubbles cause the colloids to become buoyant and rise to the air–liquid interface.^[29]^ The colloids generate bubbles in a cyclical manner that follows a pattern of bubble growth followed by bursting, causing the them to self-propel at the air–liquid interface.

In the following sections, we begin by describing experiments in which we used the colloids as microrobots to precisely manipulate passive spheres both at air–liquid interface and on the glass substrate. We then describe the bubble generation and dynamics of the bubble-propelled colloids. Then we discuss possible mechanisms that determine the bubble size and frequency of bursting. This is followed by a description of additional interesting observations of active colloid behavior, and lastly we end with a discussion of our results and possible future experiments and applications.

### 2.2. Magnetic Control

In the absence of an applied magnetic field, the active colloids tended to undergo circular trajectories, although some colloids demonstrated motion both to the right and to the left relative to their orientation, therefore performing trajectories resembling a persistent random walk. Such non-linear motion of bubble-propelled colloids has been observed previously.^[21,30]^ In Reference [30], it was found that active colloids with a small region of catalytic surface coverage displayed more persistent motion than those with a hemispherical or complete surface coverage. In our experiments, we applied magnetic fields to control the direction of motion of all of the active colloids. This allowed for the controlled manipulation of passive objects as well as maintaining the active colloids in the field of view for longer periods of time to obtain better statistics in our analysis of their behavior.

The magnetic system was custom made and is capable of generating uniform magnetic fields along any direction in the x-y plane with fields strengths at the sample’s center up to approximately 10 mT, although generally values of around 5 mT were used when guiding the microrobots. Electromagnets on opposite sides of the sample applied superimposing magnetic fields that resulted in a uniform field over the region of interest with negligible field gradients, therefore no appreciable magnetic forces were applied to the colloids. Custom made Matlab code was used to control the strength and direction of the magnetic fields generated by the electromagnets. The code was integrated with an Xbox controller, and a joystick on the controller was used to adjust the magnetic field strength and direction quickly and easily, providing a responsive system that allows for directing the active colloids while observing them in real-time. More details of the code and electromagnetic system are provided in the supporting information and an analogous system is described elsewhere.^[31]^

### 2.3. Manipulation and Assembly

**Figure 3.**
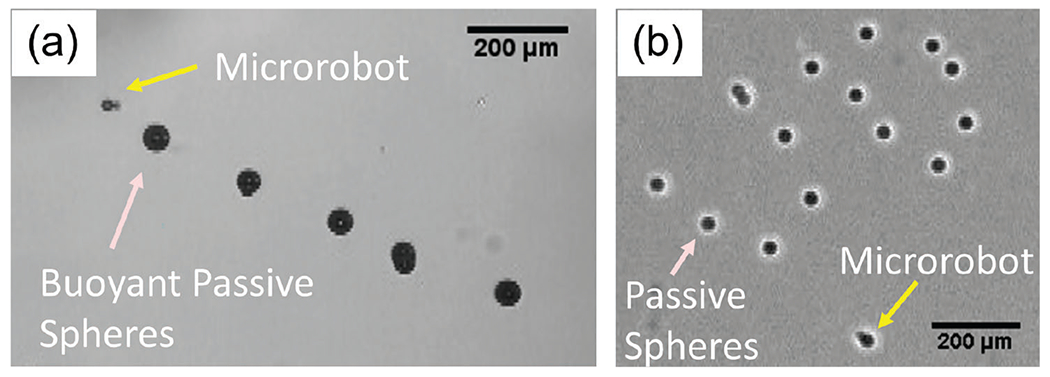
a) A bubble-propelled microrobot assembled buoyant 45–85 μm diameter spheres into a linear shape at the liquid–air interface. The microrobot is magnetically steered and is coated with platinum by e-beam deposition at a glancing-angle of ten degrees. The concentration of hydrogen peroxide was 30% b) Images showing manipulation of passive 24 μm diameter spheres into the letters “UD” on the glass substrate by a microrobot. The microrobot is coated in iron at normal incidence and then with platinum at a 20 degree glancing angle. The concentration of hydrogen peroxide was 20%.

We demonstrate magnetic control of the the active colloidal microrobots by forming a pattern with passive buoyant hollow 45–85 μm diameter spheres which float at the liquid–air interface. We are able to be assemble the hollow spheres into shapes by the microrobots, as shown in Video S1 (Supporting Information) and Figure 3a. The spheres are manipulated by directing the microrobots such that they collide with the spheres, or by placing them in near proximity to the spheres so that the fluid flow generated by the moving microrobots would exert a force on the spheres. We also demonstrate precise manipulation of smaller 24 μm diameter spheres on the substrate (see Video S2, Supporting Information and Figure 3b). Manipulation at the substrate was made possible by using a thin layer of liquid on the glass slide, so that the buoyant microrobots could physically interact with the spheres on the substrate.

### 2.4. Colloid Velocity and Bubble Production

**Figure 4.**
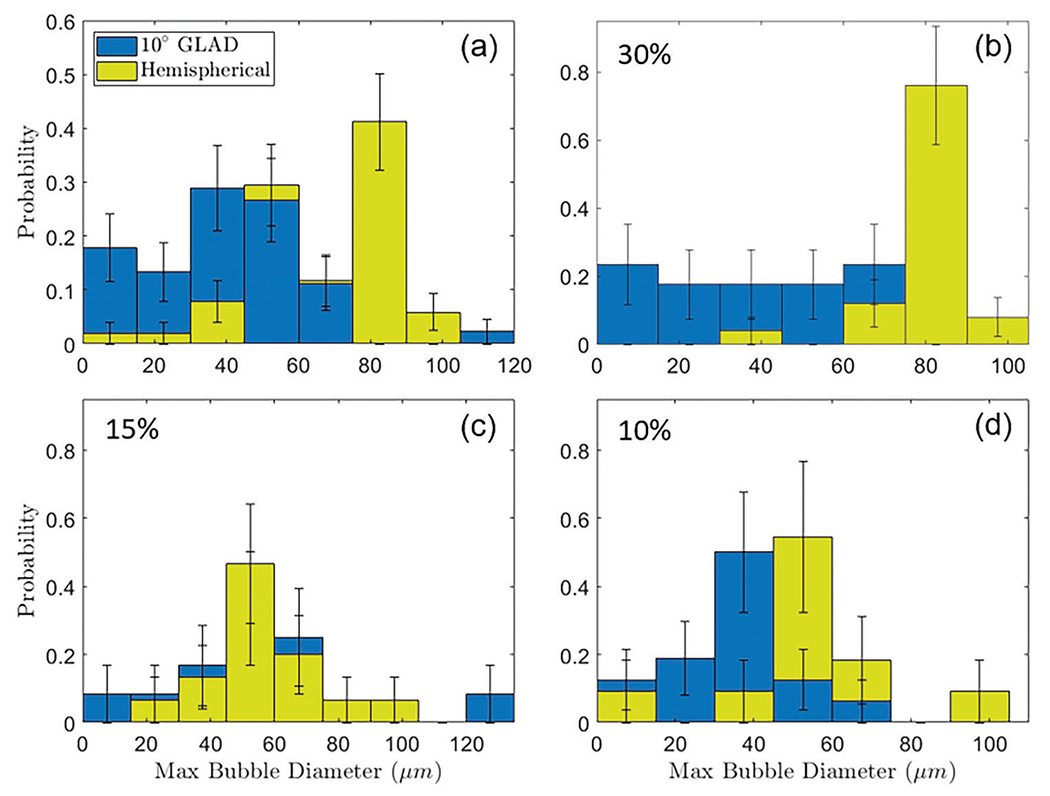
Probability of a microrobot producing a bubble of a given diameter for both the patchy and hemispherically coated microrobots for a) all hydrogen peroxide concentrations, b–d) concentrations of 10, 15, and 30%, respectively. For viewing both data sets at the same time, the larger bars are placed behind the smaller. At least ten active colloids from at least three different experiments were included in each of the six data sets (exact numbers given in the Supporting Information). The error bars correspond to square root of n uncertainties.

To quantify the distribution of maximum bubble size produced by the catalytic colloids, we calculated the probability distribution and display a histogram in Figure 4 for each of the hydrogen peroxide concentrations used in the experiments. As can be seen from the figure, the patchy colloids generally produce bubbles of smaller size than those produced by the Janus colloids, particularly at the highest concentration of hydrogen peroxide of 30%. Indeed, a subset of the patchy colloids produce bubbles of diameter similar to or smaller than the diameter of the colloids themselves. This could be a useful feature when precise control of the placement of the colloids is desired in microrobotic applications, while avoiding disturbances created by the physical interference of larger bubbles and the hydrodynamic influence created upon their bursting. The larger variability in maximum bubble size of the patchy compared to the hemispherically coated colloids could be due to slight differences in the shape or surface area of the platinum coating on the patchy colloids. Such differences could partly be due to the spheres not forming a perfect close-packed monolayer prior to deposition, differences in deposition orientations relative to the hexagonal structure, and polydispersity of sphere sizes.

**Figure 5.**
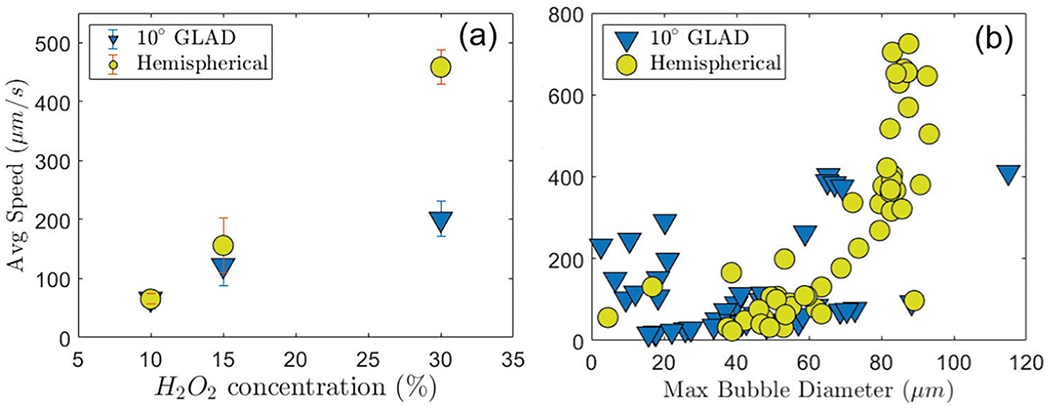
a) Average speed of the patchy (blue triangles) and Janus (yellow circles) bubble-propelled colloids as a function of hydrogen peroxide concentration. b) The average speed of the colloids versus their maximum bubble diameter, for all hydrogen peroxide concentrations. Error bars represent standard error of the mean.

One possible explanation for the decreased bubble size observed in the case of the patchy compared to the Janus colloids could be the reduced platinum coating, resulting in a lower oxygen production rate. If this is the case, one would then expect that the Janus colloids would exhibit similar behavior as the patchy colloids if their reaction rate was reduced sufficiently, for example by placing them in a solution with a lower concentration of hydrogen peroxide. As can be seen from Figure 4, lowering the hydrogen peroxide concentration does result in a reduction of the bubble size of the Janus colloids, although the probability for the smallest bubble sizes (the two left-most bins, for example) remains lower than that of the patchy colloids. We also find that the speed of the Janus colloids decreases substantially as the hydrogen peroxide concentration is decreased, as shown in Figure 5a. Further reducing the hydrogen peroxide concentration to 5% resulted in velocities that were even further reduced and no longer sufficiently larger than drift velocities to be accurately measured. We discuss the determining factors of the bubble size and frequency for these colloids in Section 2.6.

We also find that the speed of the colloids generally increases with the maximum size of the bubble produced, see Figure 5b, up to a maximum diameter of about 100 μm at which point the speed appears to have little to no dependence on bubble size. Colloids that produce bubble diameters of less than about 25 μm tend to have relatively large speeds compared to those that produce somewhat larger bubbles of 25–50 μm in diameter. This indicates a possible distinction in their bubble production/burst or propulsion mechanisms, which we will discuss later.

### 2.5. Active Colloid Dynamics

**Figure 6.**
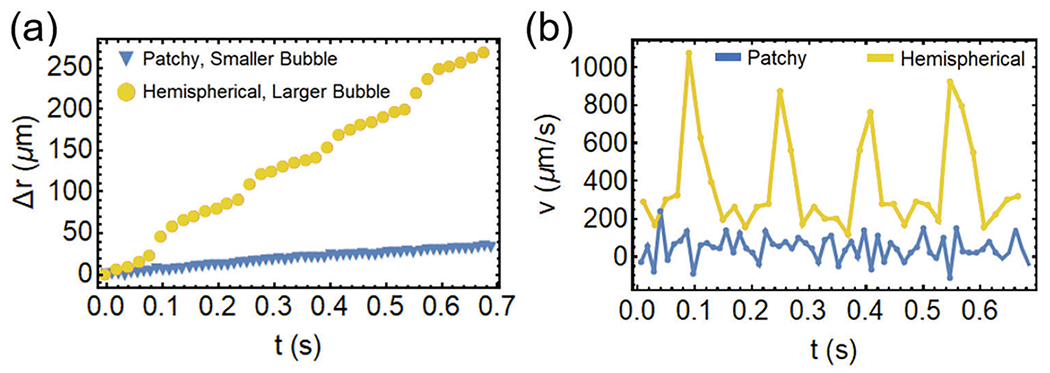
a) The distance traveled by a hemispherically coated Janus (yellow circles) and patchy (blue triangles) colloid as a function of time. b) the corresponding instantaneous velocity of the colloids. The standard deviation of the velocities of the patchy and hemispherically coated colloids are 68 and 251 μms^−1^, respectively. For the examples shown, the maximum bubble diameter of the Janus colloid was about 90 μm, the frequency of bubble production was about 5 Hz, and the concentration of hydrogen peroxide was 30%. The maximum diameter of the bubble produced by the patchy colloid was about 17 μm, the frequency was >19 Hz, and the hydrogen peroxide concentration was 10%.

The patchy microrobots generally display three types of motion. Colloids that produce relatively large bubble sizes of roughly *D*_max_ ≳ 25 μm either display exclusively forward motion with a periodic nature or a quasi-oscillatory behavior, as has been described in References [21, 22] (see Video S3, Supporting Information). However, for the colloids that produce small bubbles of approximately *D*_max_ ≲ 25 μm, this oscillation is not observed and their motion is of a much smoother nature (see Video S4, Supporting Information). This is evident from the distance travelled Δ*r* shown in Figure 6a. Figure 6a shows the difference in smoothness between the trajectories ofa 25 μm patchy colloid, which had a maximum bubble size of about 17 μm and a frequency > 19 Hz and a 25 μm Janus colloid, which had a maximum bubble size of about 90 μm and a frequency of about 5 Hz. The corresponding instantaneous velocity for the two colloids is shown in Figure 6b. The motion displayed by the Janus colloid results in periodic spikes in its measured velocity, whereas the patchy colloid that generates a smaller bubble displays a much smoother motion. The fluctuations from the mean in the case of patchy colloids is likely due to noise as there is no periodicity in the data. We find that this anti-correlation between bubble size and frequency is a general trend (see Figure S5, Supporting Information), particularly in the cases of maximum bubble diameter *D*_max_ ≲ 25 μm in which the frequency was typically greater than 10 Hz.

In general there are several forces acting on the colloid during the periodic bubble growth and burst phases. The forces that act in the horizontal direction have been identified as consisting of two forward propulsive components, *F*_growth_ and *F*_jetting_, and two backwards components, *F*_drag_ and *F*_cavitation_.^[21,22,25]^ The forward propulsive growth force, *F*_growth_, is due to the growth of the bubble, which acts to push the colloid away from the bubble. The other propulsive force, *F*_jetting_, is due to a hydrodynamic jetting flow that occurs upon the burst of the bubble.^[21]^ The bubble burst also results in a cavitation force due to a reduction in pressure, *F*_cavitation_, which pulls the colloid backwards.^[22]^ There is also a drag force, *F*_drag_, that acts against the direction of motion of the colloid.

In the vertical direction, the buoyancy of the bubble results in an upward force on the colloid, which is balanced by a downward gravitational force. The bubble growth and burst can also result in periodic vertical forces on the colloid.^[21]^

The quasi-oscillatory motion that we observe has been explained previously as a consequence of the forces produced on the colloid by the bubble as it grows and then bursts.^[21]^ The motion of the colloid after bubble burst is determined by the relative sizes of the propulsive and backward forces on the colloid. Specifically, upon bubble burst, a region of low pressure is created, which can cause the colloid to move backwards, *F*_cavitation_.^[22]^ However, a hydrodynamic jetting flow can also be created in which fluid flow creates a net forward force on the colloid, *F*_jetting_.^[21]^ In the experiments of Reference [21] it was also found that the relative amount of backward to forward motion was determined by the maximum bubble size. For bubble radii less than about 0.7 times that of the colloid, the resulting motion was backwards followed by a moderate forward motion. For bubble to colloid size ratios that are between 0.7 and 1.5, the colloids either moved backward, followed by a larger forward motion, or only moved forward. For larger bubble sizes upto about 1.8 times that of the colloid, the motion was only backwards.

**Figure 7.**
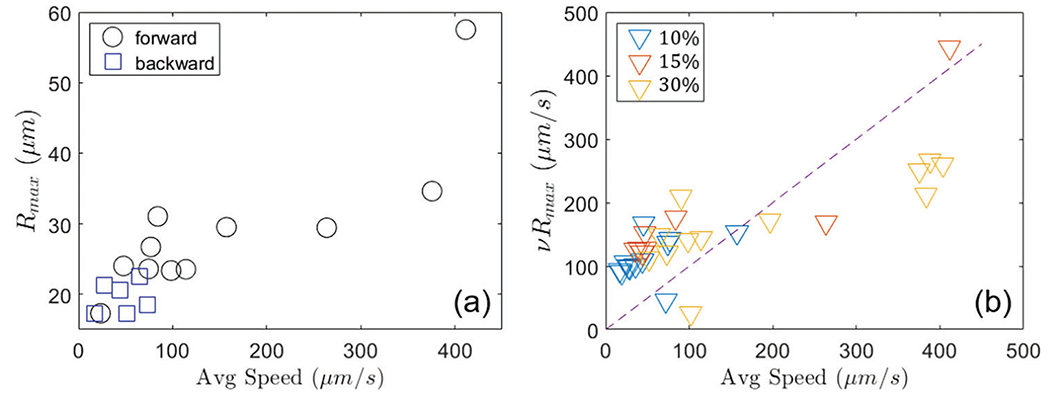
a) The maximum bubble radius versus average microrobot speed for all cases in which either a forward only (black circles) or backwards (blue squares) motion could be observed following bubble burst. b) The frequency, *ν* of the bubble growth/burst process times the maximum bubble radius, *R*_max_, plotted against the average colloid speed. The dashed line has a slope of one and corresponds to the case in which the colloid moves one bubble radius per cycle.

To investigate the forward/backward motion and its dependence on bubble size, we examined each video and separated the colloid dynamics into two categories: those in which the colloid moved backwards upon bubble burst and those in which it only moved forward. We show a plot of the maximum bubble radius, *R*_max_, versus average colloid speed in Figure 7a for the patchy colloids for cases in which the forward or backward motion could be clearly identified (cases in which the frequencies were similar to the frame rate were difficult to assess and were excluded). We also show results for the Janus colloids in Figure S3 (Supporting Information). The blue squares represent cases in which the colloid moved backward after the bubble burst and the black circles correspond to cases in which the colloid only moved forward. From the plot, one can see that instances of backwards motion occurred for colloids that produced bubbles with relatively small radii between 20–30 μm and with relatively low velocities of less than about 100 μms^−1^. Therefore, our results suggest that in the case of these colloids that produce smaller bubble sizes, the forward propulsion induced by the hydrodynamic jetting flow has a less significant influence than the backward induced motion created by the pressure decrease upon bubble burst.

Although we do observe backward motion at bubble to colloid diameter of a 1.8 ratio, we do not typically observe the forward only motion at ratios less than 1.5, in contrast to Reference [21]. This discrepancy could be due to differences in the experimental systems, for example the colloids used in that study were hollow and not at the interface. Also, the frequency of bubble creation was considerably larger in that study compared to the majority of colloids we studied. The colloid size was also somewhat larger (20–50 μm diameter compared to our 24 μm diameter). We also note that most of our videos were taken at frame rates of 30–50 fps, therefore motion that occurred on timescales less than about 30 ms would be hard to perceive in our videos.

In addition to the hydrodynamic jetting flow, another propulsion mechanism that drives the colloid forward is the growth force produced by the bubble as it expands.^[22]^ Since the bubbles produced by the colloids remain attached to the colloid surface during the growth stage, the growth force can displace the colloid forward by an amount no greater than the maximum radius of the bubble. Therefore, colloids that move with velocities in excess of *νR*_max_ , where *ν* is the frequency of bubble growth/burst and *R*_max_ is the maximum radius of the bubble, must be propelled by other mechanisms, such as the hydrodynamic jetting flow. In Figure 7b, we plot the bubble frequency multiplied with the maximum bubble radius versus the average speed of the colloid. The dashed line has a slope equal to one and separates cases in which the speed could be attributed solely to a growth force mechanism (on or above the line) from those in which additional mechanisms are required to attain the measured speeds (below the line). As can be seen from the plot, the active colloids with relatively large speeds of more than about 100 μms^−1^ move faster than what can be attributed to the growth force mechanism alone. The hydrodynamic jetting flow could account for some of the additional propulsion, and indeed the commonality of the approximate speed of 100–200 μms^−1^ at which the forward/backward transition occurred in Figure 7a and at which the data intersects the dashed line in Figure 7b implies that this may be the case. We also note, however, that the time over which the jetting flow force persisted in [21] was only around 1 ms, whereas we observe forward motion that lasts the entire duration of the bubble growth period (tens of milliseconds) in some cases. This is particularly evident for the fastest colloids. An example of this can be is shown in Video S6 and Figure S2 (Supporting Information), where it can be seen that the center of mass of the bubble itself moves forward during bubble growth at an average speed of around 300 μms^−1^ . This additional propulsion mechanism following bubble burst is unclear, but one possibility is that it is due to a self-phoretic process, like that responsible for the active motility of smaller, nonbubble propelled, microrobots, although at a surprisingly high speed. Other mechanisms that could be responsible may be capillary or Marongoni forces. Future theoretical or simulation work may be needed to help determine the cause of this forward motion of the bubble.

Another factor that can affect the resulting colloid propulsion is the orientation of the colloid relative to the bubble. In cases in which the bubble/colloid are oriented along the interface, the resulting propulsion is parallel to the interface and results in a greater velocity. We observe that the relatively slower colloids appear to reside somewhat below the bubble and therefore a component of the force likely pushes the colloid downward, like that observed in Reference [21], which would result in less efficient propulsion along the interface. We note that such a downward propulsion, if present, did not result in the focal plane of the colloid changing noticeably. Previous experimental work has shown that the application of a magnetic field along the vertical direction (perpendicular to the interface) can result in increased velocity along with decreased bubble size and increased frequency.^[32]^ It was found that this corresponds to a more steady tilt of the Janus colloid such that its face points parallel to the interface. We found that both the Janus and patchy colloids display a rotation of their catalytic surface as the bubble forms and then bursts (see. Video S5, Supporting Information). The catalytic surface of the colloid rotates upward as the bubble grows and then rotates downwards after it bursts, likely due to a competition between the buoyant torque generated on the colloid from the bubble as it grows and a gravitational torque produced by the weight of the metal cap. In the cases in which the colloid produced very small bubble sizes of less than 25 μm or so, the orientation was more steady due to the higher frequency of bubble generation. One explanation, therefore, of the relatively high speeds of the colloids that produce these small bubbles at high bursting rates compared to those with somewhat larger bubble sizes (as seen in Figure 5), is that they produce more efficient forward propulsion velocity due to a more steady and horizontal orientation of their catalytic surface. Although larger bubbles can produce more forward force due to larger hydrodynamic jetting flow, they may also tend to be less efficient because of the existence of a downwards component of the force vector.

As can be seen from Figure 5, the speeds of the active colloids increase with increasing hydrogen peroxide concentration. The relation between the two depends on how the increase in catalytic reaction rate caused by an increase in hydrogen peroxide concentration affects not only the magnitude of the forces acting on the colloids, but also on the orientation of those forces in the horizontal direction. The relative orientation of the colloid and bubble likely depends on both the bubble size and burst frequency, and possibly the gravitational forces and torques on the colloid.

### 2.6. Bubble Production Size and Frequency

In this section we will discuss observations and analysis of the bubble growth and burst. It is first relevant to determine how the hydrogen peroxide concentration relates to the reaction rate at the surface of the colloid and how this might effect the resulting bubble size. As described earlier, a bubble is formed at the surface of the patchy colloid when the concentration of oxygen produced through the catalytic reaction, reaches the saturation level of about 0.28 mol/m^3^. Specifically, the rate of oxygen production can be modeled using a Michaelis-Menten form [33],

(1) $$\alpha =\alpha_{2}\frac{{\left[H_{2}O_{2}\right]}_{V}}{{\left[H_{2}O_{2}\right]}_{V}+\frac{\alpha_{2}}{\alpha_{1}}}$$

where *α* is the rate of oxygen production, *α*_1_ and *α*_2_ are catalytic reaction rate constants, and [H_2_O_2_]*_V_* is the volume concentration of hydrogen peroxide. The ratio $\frac{\alpha_{2}}{\alpha_{1}}$ has been measured to be approximately 0.11.^[33]^ Therefore, the reaction rate of hydrogen peroxide at the surface of the colloid at a hydrogen peroxide concentration of 15% and 10% is expected to be about 0.8 and 0.5 times the rate at 30% concentration, respectively.

The smaller maximum bubble size produced by the patchy colloid as shown in Figure 4 could be attributed primarily to lower H_2_O_2_ concentrations and smaller amount of catalyst on the robot surface. Previous studies have conjectured that the maximum bubble size is generally determined by the bubble radius at which the amount of oxygen leaving the bubble through diffusion balances the amount entering the bubble via the catalytic reaction at the colloid’s surface.^[21,22]^ The amount of oxygen that is lost by the bubble increases as the surface area of the bubble increases, therefore there is a threshold maximal size at which the flux of oxygen out of the bubble becomes larger than that into the bubble and the bubble becomes unstable and can collapse. Oxygen that diffuses away from the bubble/colloid also undergoes transport due to fluid flow produced by the moving colloid and bubble growth/burst, making for a generally complex process. However, this simple flux balance model could nevertheless in principle qualitatively explain the decrease in bubble size at lower hydrogen peroxide concentrations. Also, since the patchy colloids have about 1/4 the amount of platinum on their surface, the oxygen production rate is presumably also lower, which could be responsible at least in part to the smaller bubble sizes. In addition, the small patch of platinum leads to a more localized region of oxygen concentration and hence presumably a greater gradient of oxygen concentration around the colloid. This could lead to a smaller region in which there is a sufficient amount of oxygen to sustain bubble growth, thereby result in a smaller maximum bubble size.

**Figure 8.**
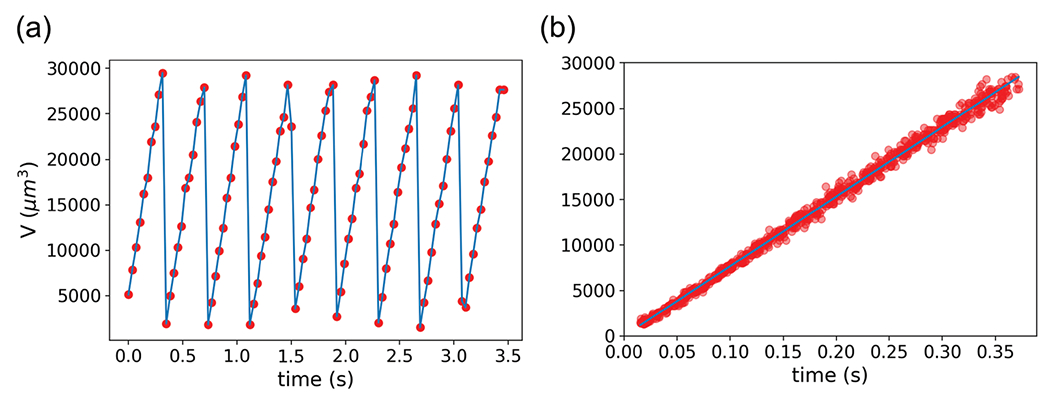
a) An example of the estimated bubble volume versus time for a patchy colloid in 30% H_2_O_2_ that produced a bubble of maximal diameter of around 39 μm at a frequency of 2.6 Hz. b) Data from multiple bubble growth/burst periods collapsed onto one curve. Also shown is a linear fit to the data.

In order to further assess whether a flux balance of oxygen is responsible for the onset of bubble burst, we estimate the volume of the bubble as a function of time. We determine the volume by measuring the 2D-image of the bubble in each frame of the video and assume a spherical shape of the bubble. Note that, for the diameters considered here, the pressure is equal to the atmospheric pressure and, to a good approximation, does not vary with bubble size .^[21]^ The volume of the bubble is therefore simply proportional to the amount of oxygen inside the bubble via the ideal gas law, *PV* = *Nk_B_T*, where *P* is the pressure, *V* is the volume, *N* is the number of oxygen molecules, *k_B_* is Boltzmann’s constant, and *T* is the temperature. We plot an example of the estimated bubble volume versus time in Figure 8a. Because of the relatively few number of data points in a given bubble growth process, we combine the data from multiple growth cycles in order to improve the temporal resolution of the data. This was done by conducting a linear fit to the data for each bubble growth cycle and shifting the data along the time-axis such that the intercepts of the linear fits passed through the origin. We show this result in Figure 8b along with a linear fit to the data. As one can see from the plot, the bubble volume is linear with time, indicating that the rate of oxygen entering the bubble is approximately constant over the lifetime of the bubble. Note that this is distinct from what was observed in Reference [21] in which the bubble radius grew more slowly than *t*^1/3^ at times approaching bubble burst. An approximately linear growth of *R*^3^ with time was observed in the case of hollow cavity bubble-propelled swimmers, however.^[34]^ The linearity we observe in the calculated bubble volume with time also suggests that the bubble does not significantly obstruct the catalytic surface as it grows, which would act to reduce the oxygen production rate over time and therefore presumably slow the growth rate of the bubble volume.

Since there is no indication of a flux balance prior to bubble burst, which would be indicated by a flattening of the data, other factors may be involved in initiating the bubble burst in our experiments, at least in cases of *D*_max_ ≳ 25 μm. We observed that hemispherically coated buoyant hollow silica spheres of diameters of 45–85 μm produced small bubbles (see Video S7, Supporting Information). If the onset of bubble burst is due to a balance of oxygen flux, one might expect that the larger surface area would result in the production of larger bubbles. Instead, the opposite is observed, which further indicates that a different process is needed to explain the bubble burst mechanism.

We note that the bubble size is too small and the frequency too large to obtain accurate data of the bubble size versus time in the case of the subset of colloids with bubble sizes of *D*_max_ ≲ 25 μm, therefore it is possible that the maximal bubble size is determined by an oxygen flux-balance in those cases. One can show that the pressure on the bubble due to surface tension, 2σ/*R* (where σ =72 mNm^−1^ is the surface tension and R is the bubble radius), starts to become important for bubble diameters of this small size.^[21]^ The surface tension is approximately equal to atmospheric pressure of 10^5^ Pa ata bubble diameter of about 3 μm. Therefore, surface tension would effect the growth of these small bubbles and would have to be taken into account.

It is also interesting to note that the volume of a stationary bubble in a liquid that is uniformly supersaturated with oxygen is expected to grow non-linearly with time.^[35,36]^ Therefore, the measured linearity of the volume of the bubble with time implies, perhaps not surprisingly given the expected non-uniform distribution of oxygen around the colloid, that this is a poor model for the process by which oxygen enters the bubble. Instead, the linearity suggests that, assuming the rate of oxygen production is constant, the rate of oxygen absorption by the bubble is proportional to the amount of oxygen produced at the colloid’s surface.

Another possible reason for the initiation of bubble burst could be due to contact of the bubble with the air interface. Such a process of bubble bursting is described in Reference [36], for example. The bubble bursting in this case is theorized to be a stochastic process. The bubble size could then be explained by the growth rate of the bubble, i.e., a slower growth rate would lead to a more probable burst occurring before the bubble has time to grow to a larger size. However, the consistency of the maximum bubble size as shown in Figure 8 implies that a stochastic process is unlikely to be responsible for the bubble bursting mechanism.

We conjecture that several other factors could be responsible for initiating bubble burst, such as detachment of the bubble from the colloid caused either by bubble buoyancy or growth force, which could cause the bubble to overcome the surface adhesive force and move away from and detach from the colloid. This in turn could lead to bubble instability and burst. These are posited in Reference [37], for example, to explain the size of bubbles in a boiling system.

The weight of the metal coating could affect the vertical tilting of the colloid during the bubble growth and burst phases, which could possibly also affect bubble size and the rate of bubble burst. As discussed earlier, the application of vertical magnetic fields has been shown to decrease bubble size and increase its burst frequency.^[32]^ We conducted preliminary experiments on Janus and patchy colloids with and without an iron coating which showed qualitatively similar behavior (smaller bubble sizes and slower velocities of the patchy compared to the Janus colloids), indicating that cap weight is not the governing factor. More statistics will be needed in future studies to determine the effect of the cap weight on the production of very small bubbles (≲25 μm) at very high burst rates, however.

The surface roughness may also effect the rate of oxygen production and, therefore, also the bubble growth and burst rate. It has been found that rougher colloidal surfaces can cause bubbles to nucleate more readily.^[29]^ Therefore, the effect of surface roughness would be an interesting future study.

### 2.7. Temperature Dependence

**Figure 9.**
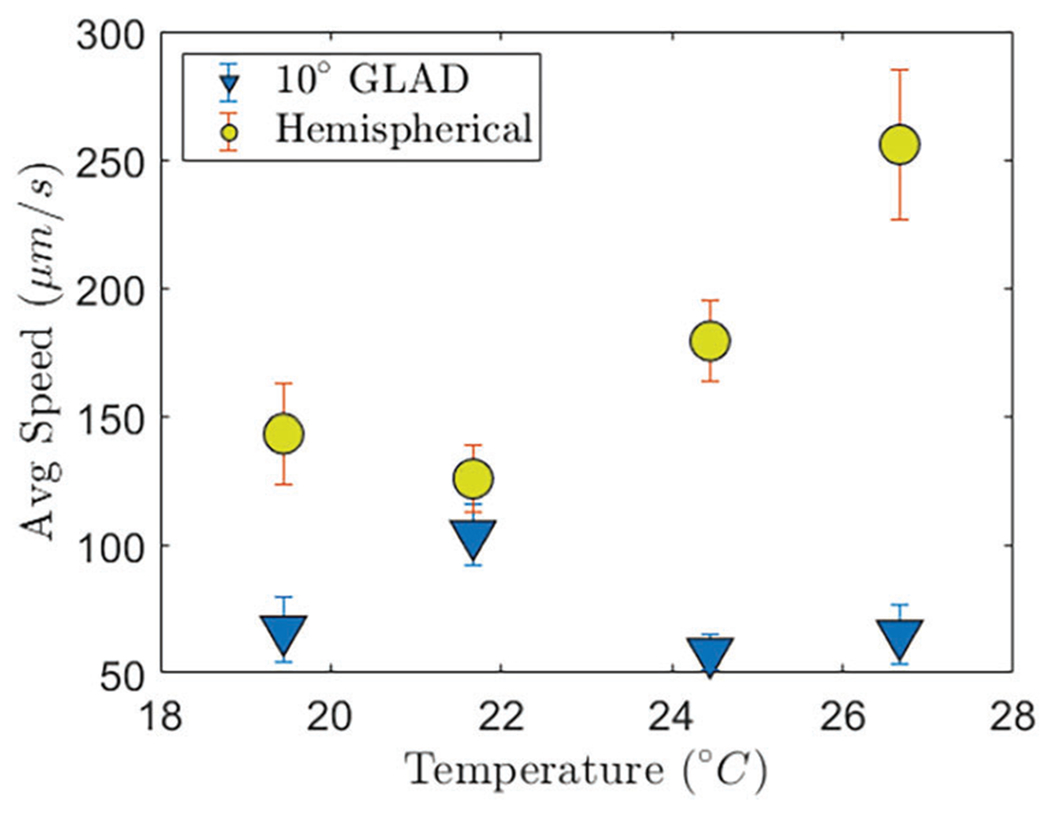
The average velocity of the active colloids as a function of temperature. All experiments were conducted at a hydrogen peroxide concentration of 30%.

We studied the temperature dependence of the colloid dynamics at temperatures of 19.4, 21.7, 24.4, and 26.7 degrees Celsius at 30% hydrogen peroxide concentration. The average velocity of the colloids at each temperature is shown in Figure 9. The average velocity of the patchy colloids shows little dependence on temperature over the temperature range studied. In contrast, the Janus colloids increase their speed by about a factor of two from 21.7 °C to 26.7 °C.

**Figure 10.**
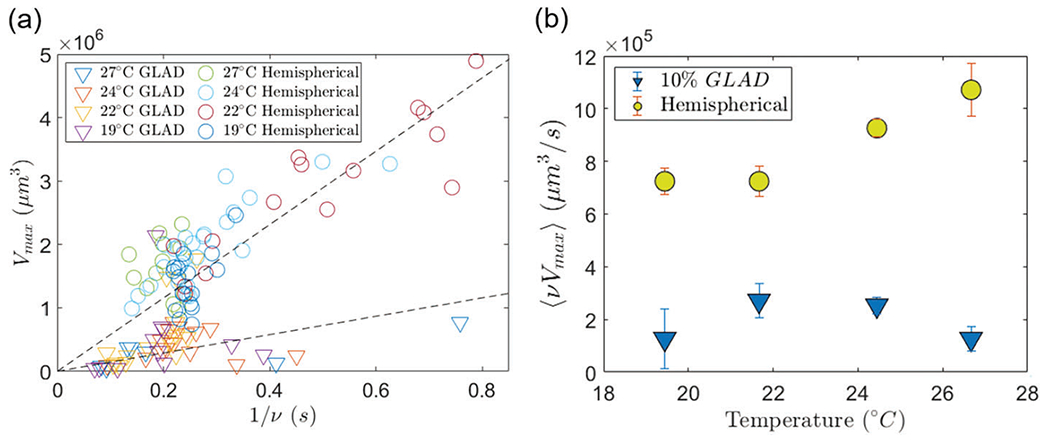
a) The estimated maximum volume of the bubble produced by a colloid versus the inverse of the bubble frequency at a range of temperatures. The dashed lines are a guide to the eye and represent constant bubble volume expansion rates. The slope of the two lines differ by a factor of four, equal to the fractional difference in platinum surface coverage of the Janus to patchy colloids. b) The ensemble median estimated maximum bubble volume times the bubble frequency, which we take as a proxy for the rate of oxygen produced at the surface of the colloids, as a function of temperature. The median rather than the mean was used to avoid outliers effecting the results. Error bars represent standard error of the mean.

To determine how the oxygen production rates depend on temperature and on the amount of platinum surface coverage of the colloids, we estimate the oxygen production rate produced at the catalytic colloids’s surface assuming that it is proportional to the rate of oxygen increase within the bubble. Because of the linear increase in bubble volume with time, as shown in Figure 8, the volume expansion rate of the bubble can simply be found from *νV*_max_ , where *V*_max_ is the maximum bubble volume and *ν* is the frequency of bubble growth. We plot *V*_max_ versus 1/ν in Figure 10a for all colloids at each temperature, and also the median of *νV*_max_ in Figure 10b. Interestingly, the relation between *V*_max_ and 1/*ν* in Figure 10a follows a fairly linear trend, which indicates that, despite the differences in maximum bubble sizes and frequencies observed in the experiments, the rate of bubble growth was fairly consistent. This implies, for example, that a smaller maximum bubble size correlates with a smaller bubble lifetime but not with a smaller or larger oxygen production rate. The two dashed lines shown in Figure 10a are guides to the eye and represent constant bubble volume expansion rates. The slopes of the two lines differ by a factor of four, which is approximately equal to the ratio of surface coverage of the Janus to patchy colloids. One can see from Figure 10b that the ratios of 〈 *νV*_max_〉 for the Janus to patchy colloids are similar to this factor across all the temperatures studied. Additionally, the bubble volume expansion rate of the Janus colloids increases by about 1.5 from the lowest to the highest temperatures. This is a similar factor to that expected from the temperature dependence of reaction rates that generally increase by a factor of about 1.4 for a change in temperature of 5 °C.

### 2.8. Interfacial Gravitaxis

**Figure 11.**
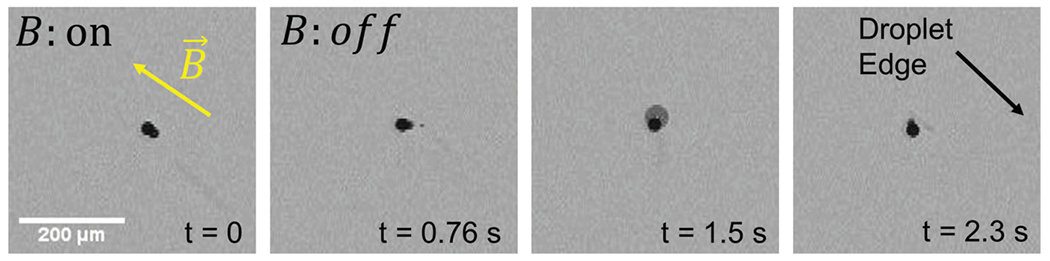
A Janus bubble-propelled colloid is first aligned with an external magnetic field (first frame) followed by zeroing of the field (second frame). The colloid rotates such that it orients towards the edge of the droplet (frames three and four). The colloid produced bubbles with a maximum diameter of about 44 μm at a rate of about 4.5 Hz.

We observe an interesting phenomenon of positive gravitaxis of the active colloids at the air–liquid interface. We notice this phenomenon generally for colloids that are near the droplet edges, presumably because of larger surface curvature of the interface near the edges. The colloids tend to orient such that they point and propel toward the edges of the droplet. An example of this interfacial gravitaxis behavior is shown in Figure 11 and Video S8 (Supporting Information), which displays an active colloid that is magnetically rotated such that it propagates away from the droplet edge and then is allowed to orient back into its preferred state facing toward the edge of the droplet upon zeroing the magnetic field. We attribute this effect to the buoyancy of the bubble creating a rotational torque on the colloid, which orients it perpendicularly to the gradient of the slope of the interface. This favored orientation results in a net propagation of the colloids down the interface and toward the edges of the droplet, thereby demonstrating behavior analogous to positive gravitaxis of Janus active colloids which orient with the heavy, coated, side facing downward.^[38]^ The colloids gather at the edge where they maintain a quasi-stationary position, presumably due to a balance of buoyant and propulsive forces.

### 2.9. Addition of Surfactant

The addition of surfactant to the solution resulted in a higher frequency of bubble production and a decrease in the size of the bubbles created by the colloid, in accordance with previous reports.^[26]^ However, it also led to the accumulation of bubbles in the solution, thus rendering this method ill-suited for micromanipulation applications where the bubbles would impede the process. A video showing the colloid in a solution with surfactant is shown in the supporting information (Video S9, Supporting Information).

## 3. Conclusion

We have studied the dynamics of GLAD coated “patchy” spherical bubble-propelled colloids at an air–liquid interface at various temperatures and hydrogen peroxide concentrations which revealed intriguing and novel behavior. We also studied Janus colloids as a control system to contrast the patchy colloids. We found that a subset of the colloids produce bubbles of very small size and that this phenomenon is enhanced in the case of the patchy colloids. Such small bubble sizes are useful for micromanipulation, which we have demonstrated with directed microassembly and manipulation of passive objects both on a substrate and at the air–liquid interface.

Additionally, we analyzed the propulsion mechanisms of these colloids using previously proposed propulsion mechanisms. It was found that they generally qualitatively account for the dynamics, however, we find that in some cases the center-of-mass of the bubble during the growth stage moves forward significantly, which has not been adequately explained previously. Future theoretical work is necessary to explore this further.

We also analyzed the bubble bursting mechanism, which revealed unexpected behavior. For example, we find that the onset of bubble burst, except perhaps for the smallest bubble sizes, is not due to an oxygen flux balance as has been proposed previously.^[21]^ We conjecture that other mechanisms, such as bubble growth force or buoyancy, may cause the bubble to detach from the colloid, leading to instability and burst. We believe that the effect of surface roughness and colloid/cap weight on the bubble growth and burst dynamics would be an interesting future study.

The patchy colloids respond less strongly to changes in both temperature and hydrogen peroxide concentrations, which can be useful in cases in which more predictable behavior is desired, for example, where environmental conditions may be unknown or not well controlled.

Aside from micromanipulation, another potential use of these magnetically controlled colloids is in interfacial applications, such as interfacial biofilm removal. It has been shown previously that the increased mixing created by bubble-propelled microrobots leads to an increase in biofilm removal efficacy.^[4]^ These buoyant active colloids could be similarly beneficial in removal of interfacial biofilms. Utilizing the magnetic guidance of these colloids could also provide for more targeted treatment.

Finally, we observe that the colloids display a positive gravitaxis toward the edges of the droplet, which we attribute to opposing downward and buoyant forces on the colloid that leads to a torque that aligns the colloid/bubble system normal to the gradient of slope of the interface. This causes the colloid to orient such that its directed motion is toward lower regions of the droplet. Such an interfacial gravitaxis has not yet been reported to the best of our knowledge.

We also note that the glancing-angle deposition could more generally be useful for coating multiple different materials on the surface of the colloids by rotating the substrate between depositions, making them potentially motile in a variety of media or able to perform multiple tasks at once. For example, adding a patch of titanium dioxide or ferrous iron to the surface would allow the colloids to serve as environmental remediation agents.^[39–41]^ As an example, we coated a layer of Pt at 20° GLAD followed by 20° GLAD of gold after a 180° substrate rotation in order to produce a coating of two different active catalysts on different sides of the colloid (see Figure S10, Supporting Information for SEM and EDX images of colloids). We confirmed that these colloids moved in hydrogen peroxide (see Video S10 Supporting Information).

The GLAD technique could also be used to create anisotropic surface coatings on the colloids. This would presumably lead to rotational torques along with translational forces, and hence produce helical motion. Well-defined helical motion has previously been sought after using smaller, diffusiophoretically propelled, colloids,^[42]^ but could be interesting to attain in bubble-propelled systems as well.

## 4. Experimental Section

### Catalytic Colloid Fabrication:

The catalytic colloids were fabricated using 24 μm diameter fluorescent magnetic polystyrene spheres (Spherotech Cat. No. FCM-20052-2) using an e-beam vapor deposition procedure. First, the spheres were pipetted into a vial and diluted bya factor of 30 in DI water. The vial was then vortexed and 30 μL of the spheres were pipetted onto the underside of a clean microscope slide. Multiple 30 μL droplets were placed on the bottom of the slide while maintaining space between each droplet to avoid merging of the droplets. The spheres sedimented and collected at the bottom of the droplets, forming a dense monolayer with only small gaps between spheres. The droplets were then allowed to evaporate, which left only the spheres on the glass surface. A 40 nm layer of platinum was deposited at an angle *θ*, as depicted in Figure 2b. Angles of *θ* = 10° and *θ* = 90° were used, resulting in two types of active colloids: those with a patch of platinum on their surface and those with hemispherical surface coverage, respectively. The Janus spheres were first coated with a 20 nm layer of iron to make them more strongly magnetic. Two of the micromanipulation videos shown were performed with patchy colloids that were produced using a glancing angle of 20°, although these were not used in any of the experiments from which data was acquired. The colloids were removed from the glass slide by washing the slide with DI water and allowing the run-off to collect in a vial until the vial was filled with approximately 500 μL of water.

### Microscope and Camera:

An Axioplan 2 upright microscope was used with an Axiovert 503 mono camera for viewing and recording videos of the colloids. Videos were acquired using a 5× objective (Zeiss) and a frame rate of about 30 fps was generally used, although occasionally higher frame rates of up to about 100 fps were utilized in order to capture greater temporal resolution of the microrobot and bubble dynamics.

### Experimental Procedures:

Experiments were conducted with glass microscope slides, which were cleaned with IPA and Acetone and then plasma cleaned for 1 h. This ensured that the glass surface was hydrophilic, resulting in the hydrogen peroxide solution spreading evenly on its surface with only a small surface curvature. Having little surface curvature was important in order to minimize the component of the buoyant forces on the colloids tangent to the interface. The vial containing the colloids was vortexed and then 1.5 μL was extracted and pipetted into a solution of 30% hydrogen peroxide, resulting in typically between 3–5 active colloids in the solution. For experiments in which a lower concentration of peroxide was used, the colloids were first placed in a 30% (w/v) solution of hydrogen peroxide for 30–45 min prior to diluting and pipetting onto the hydrophilic glass slide. The glass slide was cut into a 1 inch by 1 inch square and placed under the microscope onto a custom-built stage that contains an array of four home-built electromagnets, arranged along the*x* and *y* axes.

The colloids in 30% hydrogen peroxide solution tended to reach a steady-state behavior more rapidly and did not require the full 30 min incubation period to do so. Such initial activation of active catalytic colloids has been reported previously.^[43]^ A total volume of 140 μL of the hydrogen peroxide solution containing colloids was used in all experiments except those in which the colloids were used to move passive spheres on the substrate. In this case, only 70 μL total solution was used and time was allowed for some of the liquid to evaporate before conducting experiments. Based on the volume of liquid, we estimate that the thickness of the liquid before evaporation is approximately 220 μm and 110 μm when using 140 μL and 70 μL of the solution, respectively. To quantify the amount of evaporation, we measured the mass of 140 μL 30% hydrogen peroxide solution containing the usual amount of active colloids as a function of time at room temperature (23.9° C) and a relative humidity of 35%. It was found that over a period of an hour, the mass of liquid decreased following an exponential form with a time constant of approximately 146 min. Therefore, after a half an hour it decreased by about 20% of its initial value and by about 30% after an hour.

We also measured the concentration of hydrogen peroxide over time (more details given in the supporting information) and found that the decomposition due to the catalysis from the active colloids was negligible. We measured a small increase in concentration of hydrogen peroxide over time due to evaporation of the liquid drop, however, we expect this to have a minimal effect on their behavior during the experimental time frame.

Manipulation of passive spheres at the air–liquid interface was conducted by adding hollow glass microspheres (0.21 g/cc density) with a diameter of 45–85 μm (Cospheric) to the hydrogen peroxide solution containing the active colloids.

It was found that the active colloids exhibit a wide range of bubble sizes, bubble burst frequencies, and velocities. We hypothesize that this was due to variability in the manufacturing process. Despite this variability, any given active colloid generally produced bubbles at a remarkably consistent rate and size.

### Colloid Behavior Prior to Steady-State:

On occasion, time was required for the colloids to reach their maximal bubble production rate after adding them into the peroxide solution and observing them on the slide. Therefore, videos were acquired once the colloids reached a steady-state in which they displayed consistent behavior and approximately maximal speed. It was observed that the colloids began generating bubbles soon after adding them to the substrate at 30% hydrogen peroxide concentration but took longer to reach a steady-state at lower concentrations. The time required was generally approximately 30 min, although occasionally a longer waiting time was needed at the lower hydrogen peroxide concentrations.

Occasionally some colloids produced large bubbles that continued to grow without bursting for an unusually extended period of time, on the order of a minute or so. This was particularly prevalent in the first several minutes of adding the colloids to the glass slide before reaching steady-state. Due to buoyant forces, these colloids aggregated near the center of the slide, where the interface is at its highest point.

### Temperature Control:

The temperature of the environment was controlled by enclosing and heating the lab space around the microscope. A hair dryer was used to supply heat and the temperature was measured using a digital thermometer near the microscope. Experiments were conducted within 0.5 degrees of the values stated.

### Video Analysis:

Image analysis of the colloid’s speed and the bubble size and frequency of growth was performed using custom python code, details of which are provided in the supporting information. Briefly, image thresholding was performed to locate the colloid and bubble center of mass and determine the bubble size. The frequency of bubble growth/dissolution was found by computing a fast-Fourier transform of the bubble size as a function of time.

### Statistical Analysis:

Histograms show probability based on the number of counts in each bin divided by the total number of counts. The error bars estimate the uncertainty in the probability based on square-root of n uncertainties, i.e., by taking the square-root of the number of counts in each bin divided by the total number of counts. The total number of counts was at least ten from at least three different experiments. The total numbers for each experiment are provided in the supporting information. Error bars in plots that show averages were based on the standard error of the mean, i.e., the standard deviation of the data divided by the square-root of the total number of data. Data shown in Figure 10b and Figure S1b (Supporting Information) are based on the median rather than the mean to suppress the effect of outliers skewing the data. Video analysis was performed in Python and analysis of data was performed in Mathematica and Matlab.

## Supplementary Material

video 1

video 2

video 3

video 4

video 5

video 6

video 7

video 8

video 9

video 10

supporting information

Supporting Information is available from the Wiley Online Library or from the author.

## Data Availability

The data that support the findings of this study are available from the corresponding author upon reasonable request.
