# Supplementary material for: Patchy Bubble-Propelled Colloids at Interfaces
Source: Adv Mater Interfaces. Author manuscript; Available in PMC 2026 Jun 11. (PMC13251815; doi:10.1002/admi.202300226)
Supplement: supporting information [file NIHMS2178210-supplement-supporting_information.docx]

Supporting Information

**Patchy Bubble-Propelled Colloids at Interfaces**

David P. Rivas, Max Sokolich, Harrison Muller, Sambeeta Das

*Corresponding author. Email: [samdas@udel.edu](mailto:samdas@udel.edu)

**This PDF file includes:**

Supplementary Text

Figs. S1 to S11

Tables. S1 to S2

Movies S1 to S10

References (2)

Supplementary Text

Magnetic Control

We used custom Matlab code integrated with an NI-DAQ control system to adjust the current going to each of the electromagnetic coils, and hence control the strength and direction of the magnetic field (see Fig. S9). An X-box joystick was used to communicate with the Matlab code using the HebiJoystick library. The code was similar to that described in SI Ref. (1).

Tracking and Analysis

Tracking of the colloids as well as determining their bubble size and frequency of growth was performed using custom python code. The code first applies a thresholding which produces a binary image. For brightfield captures, the background was first subtracted from each image. The colloids were located by finding the center of mass of each connected region. Trajectories were determined after associating this location data with a particular colloid, which was done using the “link” function in a particle tracking toolkit called “trackpy” (2). The colloid speed was found after smoothing the x and y data followed by fitting an interpolation function using scipy’s univariate-spline interpolation algorithm with default settings. The interpolation function was smoothed prior to taking a time-derivative, giving a velocity in the x and y directions. The speed was then found from the square-root of the sum of the x and y velocities squared. Since the center of mass that was found after thresholding generally lies between the center of the bubble and the colloid, the smoothing of the center of mass data not only reduces noisy artifacts, but also eliminates the propagation of this localization error into the results of the colloidal speed. The bubble size was determined by finding the area of the connected region after thresholding and subtracting out the estimated area of the colloid in the absence of a bubble. The maximum bubble size was determined by finding the maximum size in multiple segments of the video, then taking a median of these values. This automated method was found to be robust, although not very precise for very small bubbles. For bubbles with a maximum size similar to that of the colloid or smaller, the maximum size was measured by hand. The frequency of the bubble growth was determined by taking a fast-Fourier transform of the bubble size as a function of time and selecting the frequency with the highest amplitude. At least 10 colloids were recorded in at least three different experiments to determine the statistical values provided in the figures.

Probability distributions of bubble sizes were calculated by binning of the data and normalizing by the total number of data points. The uncertainties in the probability were found by taking the square root of the total number of data points in each bin and dividing by the total number of data points.

Other Forces and Torques

We do not observe the focal plane of the colloids to change during their motion, despite the change in buoyancy of the colloids as the bubble grows and then bursts. One can show that the expected sedimentation velocity of the colloids in the absence of a bubble is small enough that, for the typical frequencies of bubble production of 2-5 Hz, the colloids would not sink noticeably, in agreement with our observation.

Fluorescence imaging revealed that the colloids wobble during each cycle, likely due both to the torque generated on the colloid as the bubble grows and then bursts and to the rotational torque produced by the weight of the metal cap (see Vid. 3).

Temperature Control

The temperature-controlled experiments were conducted approximately 5 months after the hydrogen peroxide-controlled experiments, therefore the longer time between their fabrication and use or the differences in environmental conditions could be responsible for the somewhat lower speed of the colloids in the temperature-controlled experiments compared to those in the hydrogen peroxide controlled experiments at nominally similar temperatures and concentrations of peroxide. We speculate that this is due to a lower efficiency of the catalytic surface of the colloids used in the temperature-controlled experiments, resulting in a slightly reduced oxygen production rate. In the temperature-controlled study, the colloids also required a somewhat longer time to reach maximal velocity. The qualitative behavior of the colloids at steady-state in the two experiments was maintained, however.

We also note that although the decomposition of hydrogen peroxide is an exothermic reaction, due to the low quantity of micromotors in the solution, the reaction rate is very low. We measured the temperature of a 3 mL 30% hydrogen peroxide solution with the usual concentration of active colloids in a glass vial over the period of an hour and found no change from its initial value.

Hydrogen Peroxide Concentration Over Time

We measured the hydrogen peroxide concentration as a function of time to ensure that it remained consistent throughout the experimental time frame. We used a method of titration to determine the amount of hydrogen peroxide with the solution. We added 35 $\mu L$ of 30% hydrogen peroxide to several plastic tubes along with 0.6 $\mu L$ of the active colloids and measured the concentration of each tube at various times. To determine the concentration, we pipetted NaOCl into the H_2_O_2_ solution. Adding the NaOCl resulted in a great number of bubbles forming until a critical amount was added at which point no vigorous bubble creation was observed. The reaction of NaOCl produces oxygen bubbles and salt. Once an equal amount of moles of NaOCl and H_2_O_2_ are present in the solution, the reaction stops. Using this method, we found that the hydrogen peroxide concentration did not decrease measurably even after 5 hours. It was still about 83% of its initial value after 19.5 hours. Assuming a linear decrease with time, we estimate that the concentration of hydrogen peroxide decreased by only about one hundredth of its initial value over a time period of 1.5 hours. This indicates that the decomposition of hydrogen peroxide due to the catalytic colloids was negligible over the relevant experimental timeframe.

We also measured the concentration of hydrogen peroxide after letting it evaporate on a glass slide to about 69% of its initial volume and found an increase from 30% to 37.5 $\pm$ 4 %. The increase by a factor of 1.25 is less than the factor by which the volume decreased (1.44), indicating that the amount of H_2_O_2_ decreased somewhat over time. The reason for this could be due to evaporation or decomposition into water and oxygen which may occur at a greater rate on the glass slide than in the plastic tube that we used in the experiment discussed above. Therefore, we conclude that although the concentration of hydrogen peroxide may increase by a small amount due to evaporation, it was fairly consistent throughout the experimental timeframe.

Bubble Volume Expansion Rates Versus Hydrogen Peroxide Concentration

Similar to the temperature dependent results shown in Fig. 10, we plotted the volume expansion rates of the bubbles produced by the colloids for different hydrogen peroxide concentrations (see Fig. S1). Figure S1(b) shows that the rate at which the bubble volume grows has little dependence on the H_2_O_2_ concentration in the case of the patchy colloids. However, the Janus colloids show a large increase in bubble volume growth rate at the largest H_2_O_2_ concentration compared to the lowest two. Therefore, in both the temperature and H_2_O_2_ controlled experiments, the bubble volume growth rate of the patchy colloids is nearly constant. One possibility for this is that the patchy colloids undergo a more efficient catalytic reaction at lower temperatures and hydrogen peroxide concentrations. The distribution and thickness of the platinum on the patchy colloids is less uniform than that on the hemispherically coated Janus colloids, therefore the two types of colloids may have different catalytic properties.

Persistence Length Calculation

We calculated the persistence length of the colloid trajectory by computing the average cosine of $\theta$, the angle between vectors tangent to the trajectory as a function of distance between the points at which each tangent was calculated. This was done by first smoothing the trajectory using a rolling mean of 4 frames in order to reduce the effect of noise on the result. The tangent vectors were found by taking the difference between adjacent points on the trajectory. The cosine of $\theta$ was found by taking the dot product between the normalized tangent vectors. The data was binned according to the distance between the center of the points that were used to find the tangent vectors and the average of the cosine in each bin was determined. The resulting values were then fit to an exponential function of the form $C\left( l \right)= e^{-l/P}$, where $l$ is the distance between the points on the trajectory and $P$ is the persistence length.


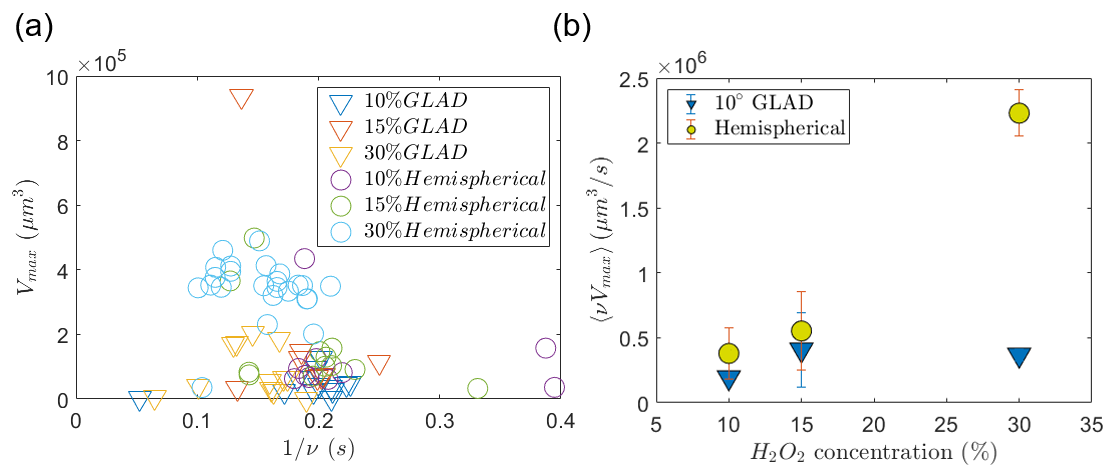


Fig. S1.

(a) The estimated maximum volume of the bubble produced by both a patchy and hemispherically coated colloid versus the inverse of the bubble frequency at the different hydrogen peroxide concentrations used in the experiments. (b) The ensemble median estimated maximum bubble volume times the bubble frequency, which we take as a proxy to the rate of oxygen produced at the surface of the colloid. The median rather than the mean was used to avoid outliers effecting the results. Error bars represent standard error of the mean.


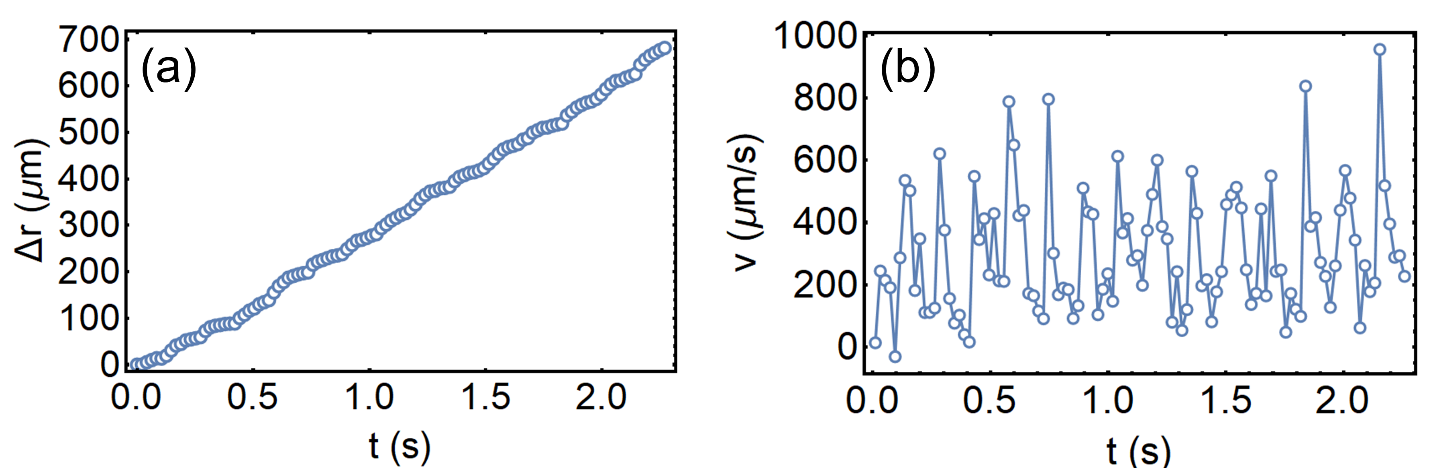


Fig. S2.

(a) The distance traveled by the center of the bubbles generated by the colloid shown in Vid. 4. (b) The instantaneous velocity of the center of the bubbles. The consistent positive values demonstrate that a forward propulsive force acts on the bubble and persists throughout the entire growth/burst cycle.


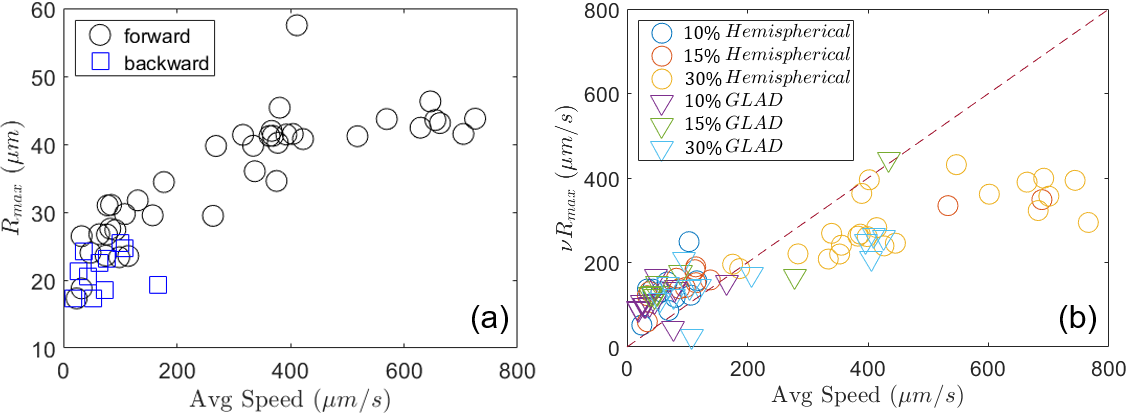


Fig. S3.

(a) The maximum bubble radius versus average colloid speed for all cases in which either a forward only (black circles) or backwards (blue squares) motion could be observed following bubble burst. (b) The frequency, $\nu$ of the bubble growth/burst process times the maximum bubble radius, $R_{max}$, plotted against the average colloid speed. The dashed line has a slope of 1 and corresponds to the case in which the colloid moves one bubble radius per cycle.


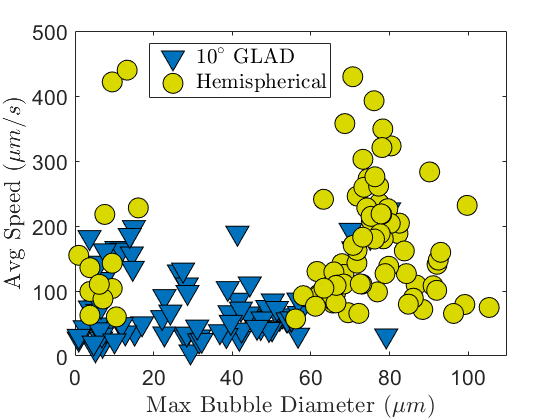


Fig. S4.

The average speed of the colloids versus their maximum bubble diameter, for all temperatures studied.


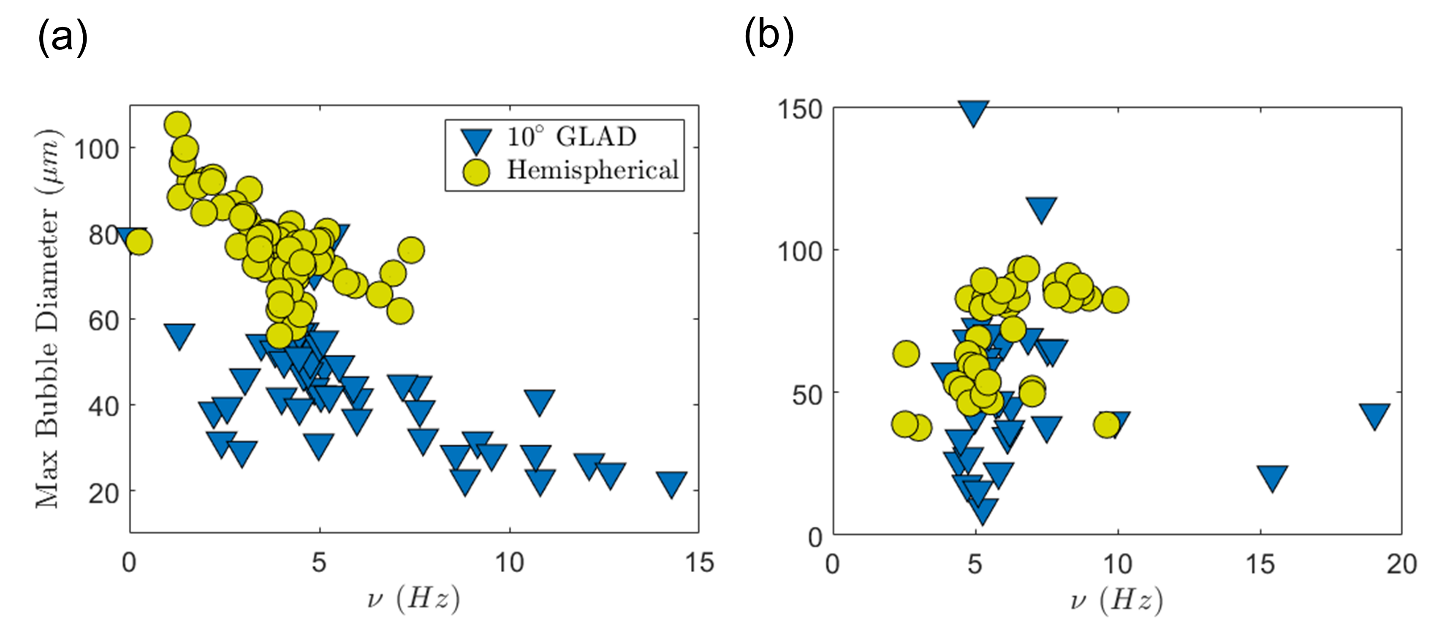


Fig. S5.

Maximum bubble diameter versus frequency for all (a) temperatures and (b) hydrogen peroxide concentration used. All cases in which the frequency was too fast to measure, which typically only occurred for colloids with $D_{max}<25 \mu m$, are omitted.


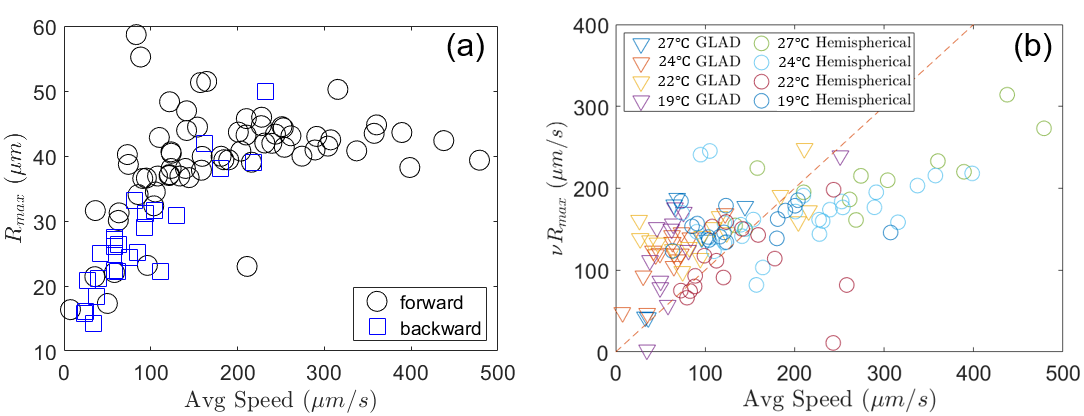


Fig. S6.

Data shown corresponds to temperature control experiments. (a) The maximum bubble radius versus average colloid speed for all cases in which either a forward only (black circles) or backwards (blue squares) motion could be observed following bubble burst. (b) The frequency, $\nu$ of the bubble growth/burst process times the maximum bubble radius, $R_{max}$, plotted against the average colloid speed. The dashed line has a slope of 1 and corresponds to the case in which the colloid moves one bubble radius per cycle.


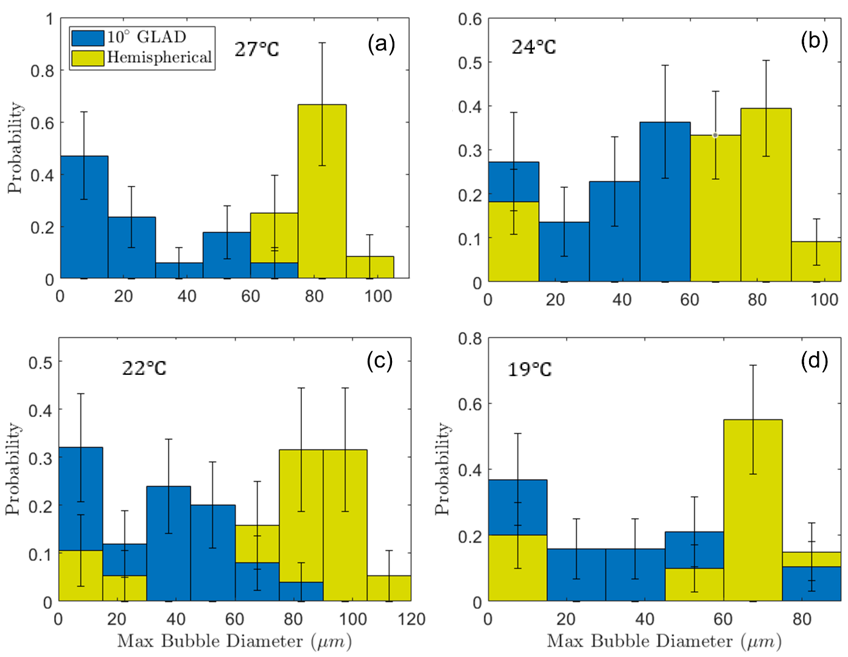


Fig. S7.

Probability of a colloid producing a bubble of a given diameter for both the patchy and hemispherically coated colloids at different temperatures of 27, 24, 22, and 19° C in (a-d), respectively. To allow for viewing both data sets on one plot, the larger of the two bars is placed behind the smaller. The error bars correspond to square root of N uncertainties.


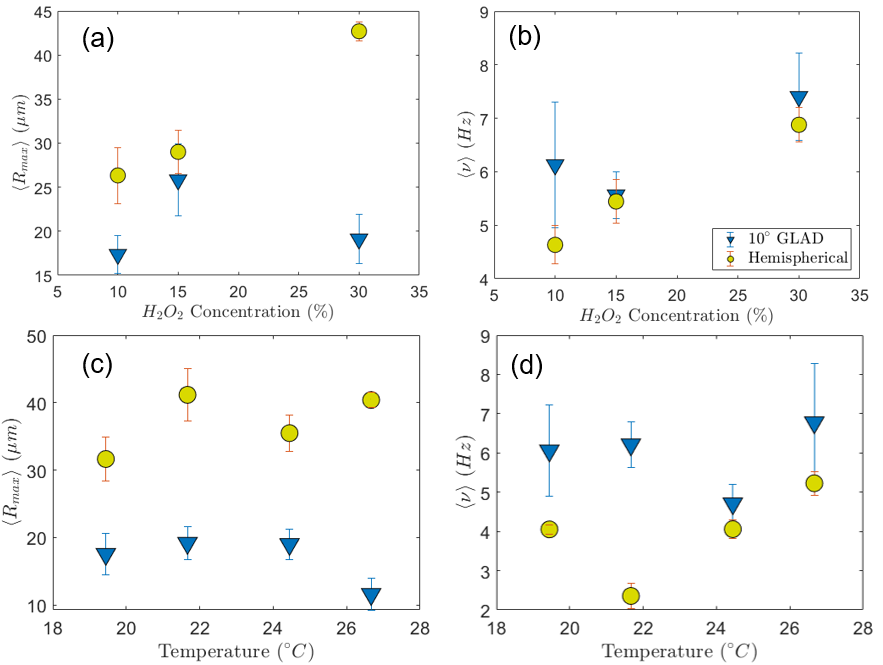
Fig. S8.

(a,b) Ensemble average maximum bubble radius and frequency, respectively, for all hydrogen peroxide concentrations studied. (c,d) Ensemble averages at each temperature used in the experiments. Note that frequencies that were too high to be measured accurately were omitted from the calculated averages shown.


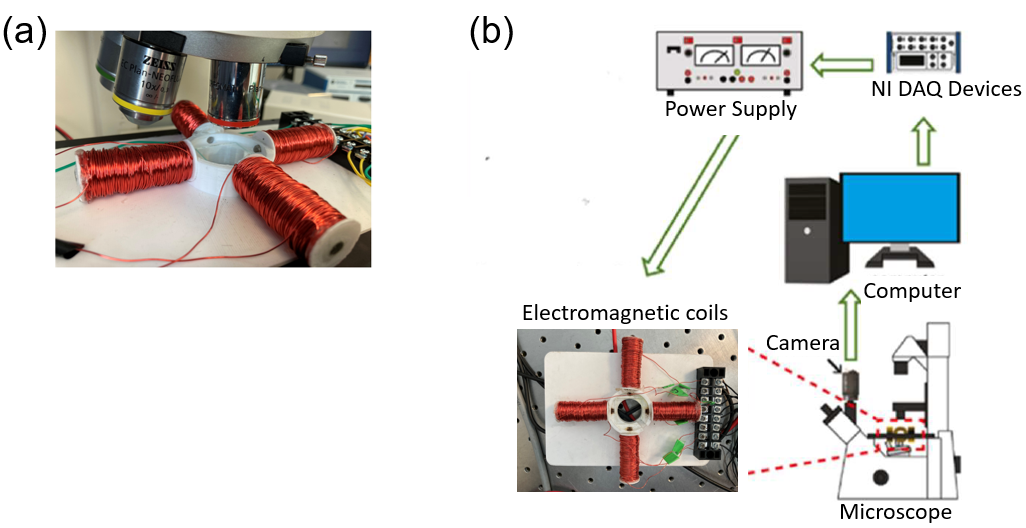
**Fig. S9.**

(a) An image of the electromagnetic coils used in the experiments. (b) Schematic diagram of the control system.


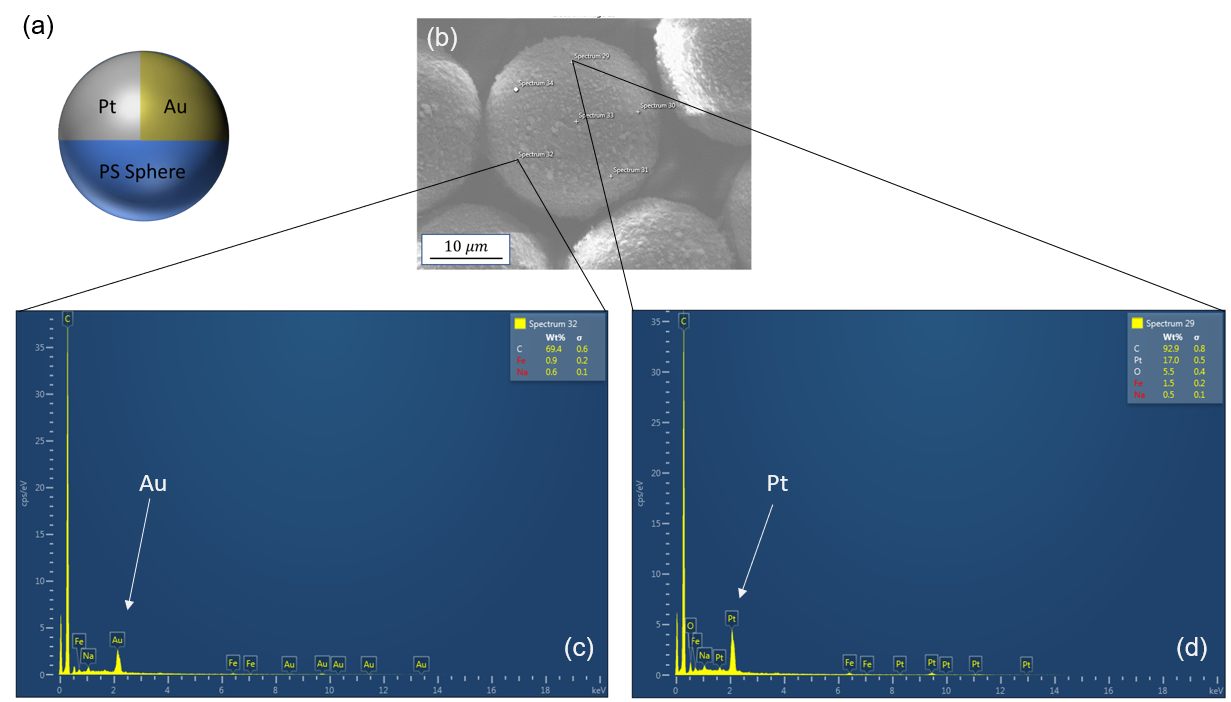
Fig. S10.

(a) Schematic of a colloid with Pt and Au GLAD coatings. (b) SEM image of the colloid. (c,d) EDS spectra on two sides of the colloid showing peak signals corresponding to Pt and Au, respectively.


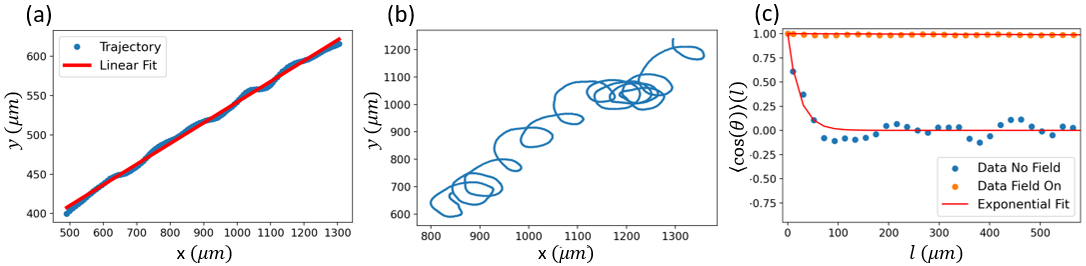
Fig. S11.

(a) An example of a smoothed trajectory of an active colloid with a constant magnetic field applied. A linear fit was performed on the data to indicate the linearity of the trajectory. The root-mean-squared residual between the linear fit and the data was approximately 3 $\mu m$. (b) An example of a smoothed trajectory of an active colloid with no field applied. (c) A plot showing the calculated average cosine of theta, the angle between tangent vectors along the active colloid trajectories as a function of distance between the points at which the tangent vectors were found. The orange data corresponds to the case with a magnetic field applied and the blue data to the case without a magnetic field. The solid red curves represent exponential fits to the data and provide calculated persistence lengths of about 23 $\mu m$ in the case of no applied field and 48 mm in the case of the constant applied field. Such a large persistence length in the latter case is expected since the colloids with a magnetic field applied do not reorient, therefore their persistence length is effectively infinite.

| Peroxide Concentration | GLAD | Janus |
| --- | --- | --- |
| $30\%$ | 18 | 32 |
| $15\%$ | 12 | 15 |
| $10\%$ | 16 | 11 |

Table S1.

The number of active colloids analyzed at each hydrogen peroxide concentration.

| Temperature | GLAD | Janus |
| --- | --- | --- |
| $26.7℃$ | 17 | 12 |
| $24.4℃$ | 22 | 33 |
| $21.6℃$ | 25 | 19 |
| $19.4℃$ | 19 | 20 |

Table S2.

The number of active colloids analyzed at each temperature.

Movie S1.

Manipulation of passive hollow buoyant spheres at the air-liquid interface by a patchy colloid microrobot to form a linear pattern (30x real-time) and a square grouping (4x real-time).

Movie S2.

Manipulation of passive 25-micron diameter spheres at a solid substrate using patchy colloid microrobots in a thin liquid layer. The videos show the arrangement of the passive spheres into the letters “UD” (30x real-time), into a circular pattern (60x real-time), and cargo carrying (10x real-time).

Movie S3.

An example of quasi-oscillatory motion of a patchy colloid, 0.25 times real-time.

Movie S4.

An example of motion of a patchy colloid that produces small bubbles.

Movie S5.

Fluorescent videos of colloids demonstrating rotation along an axis parallel to the interface during the bubble burst and growth phases. The rotation is larger for cases with larger bubble sizes with lower burst frequencies. The first video shows a patchy colloid producing bubbles at a frequency of about 6 Hz and a maximum diameter of approximately 34 microns. The second video shows a patchy colloid producing bubbles at a frequency greater than 20 Hz with a maximum diameter of approximately 13 microns. The third video shows a Janus colloid producing bubbles at a frequency of around 7 Hz with a maximum diameter of approximately 100 microns. During the videos, magnetic fields were used to steer the active colloids.

Movie S6.

An example of a Janus colloid that does not display any backward motion upon bubble burst, 0.25 times real-time.

Movie S7.

An example of the motion and bubble production of a hollow 65-micron diameter Janus colloid, 0.25 times real-time.

Movie S8.

Videos showing active colloids near the edges of the liquid droplet propagating towards the edges (2x real-time). The second video shows a colloid that is magnetically rotated to move away from the edge and then allowed to rotate freely upon zeroing of the field, which results in the colloid rotating and swimming back towards the edge.

Movie S9.

An example of a magnetically steered active colloid in a solution with surfactant and tracer particles showing the production of a large quantity of relatively small bubbles (4x real-time).

Movie S10.

Motion of patchy colloids that have a GLAD 20-degree coating of platinum and gold on opposite sides of the colloid.

**References**

1. Sokolich, M., Rivas, D., Yang, Y., Duey, M. & Das, S. ModMag: A modular magnetic micro-robotic manipulation device. *MethodsX* **10**, 102171 (2023).

1. D. B. Allan, T. Caswell, N. C. Keim, C. M. van der Wel, R. W. Verweij, soft-matter/trackpy: Trackpy v0.5.0 (2021).
